# Supplementary material for: Barriers and facilitators to successful management of type 2 diabetes mellitus in Latin America and the Caribbean: A systematic review
Source: PLoS One. 2020 Sep 4;15(9):e0237542. doi: 10.1371/journal.pone.0237542 (PMC7473520; doi:10.1371/journal.pone.0237542)
Supplement: S1 Text — (DOCX) [file pone.0237542.s002.docx]

# *S1 Text*. Search Strategies.

(16^th^ June 2020)

**MEDLINE via Pubmed:**

Results: **221**

(("diabetes mellitus"[MeSH Terms] OR diabetes[title/abstract]) AND ("facilitator"[Text Word] OR "enabler"[Text Word] OR "barrier"[Text Word] OR "obstacle"[Text Word] OR "challenge"[Text Word] OR "difficulty"[Text Word] OR "difficulties"[Text Word]) AND ("south america"[Text Word] OR "central america"[Text Word] OR "latin america"[Text Word] OR "caribbean"[Text Word] OR "antigua and barbuda"[Text Word] OR "argentina"[Text Word] OR "aruba"[Text Word] OR "bahamas"[Text Word] OR "barbados"[Text Word] OR "belize"[Text Word] OR "bolivia"[Text Word] OR "brazil"[Text Word] OR "british virgin islands"[Text Word] OR "cayman island"[Text Word] OR "chile"[Text Word] OR "colombia"[Text Word] OR "costa rica"[Text Word] OR "Cuba"[Text Word] OR "curacao"[Text Word] OR "dominica"[Text Word] OR "dominican republic"[Text Word] OR "ecuador"[Text Word] OR "el salvador"[Text Word] OR "grenada"[Text Word] OR "guatemala"[Text Word] OR "guyana"[Text Word] OR "haiti"[Text Word] OR "honduras"[Text Word] OR "jamaica"[Text Word] OR "mexico"[Text Word] OR "nicaragua"[Text Word] OR "panama"[Text Word] OR "paraguay"[Text Word] OR "peru"[Text Word] OR "puerto rico"[Text Word] OR "saint lucia"[Text Word] OR "saint kitts and nevis"[Text Word] OR "saint martin"[Text Word] OR "saint vincent and the grenadines"[Text Word] OR "suriname"[Text Word] OR "trinidad and tobago"[Text Word] OR "united states virgin islands"[Text Word] OR "uruguay"[Text Word] OR "venezuela"[Text Word])) AND (("1950/01/01"[PDAT] : "2019/02/28"[PDAT]) AND "humans"[MeSH Terms] AND (English[lang] OR French[lang] OR Portuguese[lang] OR Spanish[lang])

**Web of Science:**

(from Web of Science Core Collection)

Results: **588**

(de Colección principal de Web of Science)

TOPIC: ((diabetes)) AND TOPIC: ((facilitator OR enabler OR barrier OR obstacle OR challenge OR difficult*)) AND TOPIC: ((saint barthelemy) OR (virgin islands) OR (caribbean Netherlands) OR montserrat OR guadeloupe OR (falkland islands) OR (french Guiana) OR Martinique OR (South America) OR (Central America) OR (Latin America) OR Caribbean OR (Antigua and Barbuda) OR Argentina OR Aruba OR Bahamas OR Barbados OR Belize OR Bolivia OR Brazil OR (british virgin islands) OR (cayman island) OR Chile OR Colombia OR (Costa Rica) OR Cuba OR curacao OR Dominica OR (Dominican Republic) OR Ecuador OR (El Salvador) OR Grenada OR Guatemala OR Guyana OR Haiti OR Honduras OR Jamaica OR Mexico OR Nicaragua OR Panama OR Paraguay OR Peru OR (Puerto Rico) OR (Saint Lucia) OR (Saint Kitts and Nevis) OR (saint martin) OR (Saint Vincent and the Grenadines) OR Suriname OR (Trinidad and Tobago) OR (united states virgin islands) OR Uruguay OR Venezuela))

Refined by: LANGUAGES: (ENGLISH OR SPANISH OR PORTUGUESE OR FRENCH)

Timespan=All years

Databases: SCI-EXPANDED, SSCI, A&HCI, CPCI-S, CPCI-SSH, BKCI-S, BKCI-SSH, ESCI, CCR-EXPANDED, IC.

**Scielo:**

Results: **407**

(diabetes) AND ("facilitator" OR "enabler" OR "barrier" OR "obstacle" OR "challenge" OR "difficulty" OR "difficulties")

**LILACS:**

Results: **379**

Mediante la Biblioteca Virtual de Salud para poder buscar por Descriptor:

Portal regional de la BVS / Lilacs

Búsqueda con términos en inglés, la literatura peer-review tiene título y abstract en inglés.

tw:(( (tw:(“diabetes mellitus”) OR mh:(”diabetes mellitus”)) AND (tw:("facilitator" OR "enabler" OR "barrier" OR "obstacle" OR "challenge" OR "difficulty" OR "difficulties")) ) AND (instance:"regional") AND ( db:("LILACS")) )
