# Supplementary material for: Barriers and facilitators to successful management of type 2 diabetes mellitus in Latin America and the Caribbean: A systematic review
Source: PLoS One. 2020 Sep 4;15(9):e0237542. doi: 10.1371/journal.pone.0237542 (PMC7473520; doi:10.1371/journal.pone.0237542)
Supplement: S2 Text — (DOCX) [file pone.0237542.s003.docx]

# *S2 Text*. Critical appraisal tool.

Possible answers: Yes / No /Unclear

ONLY THE SECTIONS THAT INTEREST TO OUR OBJECTIVES SHOULD BE FILLED

Note: Mixed methods studies must meet the quality criteria of qualitative and quantitative studies (questions Q2b and Q2c and all sections of Q4), provided that the barriers and / or facilitators of DMT2 management have been evaluated by that method.

**1. Are the research objectives clearly defined?**

- Is the research question implicit / explicit?

- Is the research objective/s clearly identified?

**2a. Is the sample (or the sample procedure) properly defined?**

- The methodology used to select the sample has been specified

- It is adequately described how it was selected (place of study, loss of potential participants (is it detailed?), inclusion and exclusion criteria)

**2b**. Only quantitative or mixed studies: **Is the study free from selection bias?**

- Consecutive patients or random sample that met inclusion criteria

- The reasons for loss are justifiable if losses are detailed and do not exceed 20% ​​of the sample

**2c**. Only qualitative or mixed studies: **Is the study free from selection bias?**

- The selection criteria are justified related to the study aim / research question.

- The decisions that have motivated your selection in an intentional sampling have been described.

- Is the reason for loss detailed?

**3. Is the methodology used to respond to the stated objectives justified? Is the collection of information adequate?**

- The methodology used to collect barriers and/or facilitators has been specified

- The methodology used to collect barriers and/or facilitators is adequate to respond to the objective

- The data has been collected directly from the participants (primary data), not from a secondary source

**4. Are the results clearly presented? Have measures been taken to make the results reliable?**

- The results answer the research question (barriers and facilitators).

- The results are presented in a detailed, understandable way.

- Only quantitative or mixed studies: the analysis is correct, the data is clearly presented in a table, the data is associated with precision measures.

- Only qualitative or mixed studies (fulfilling any of them): triangulation of the data, returning of the results to the participants and validation by them, or providing the citations that support the results

**5. Are the results applicable to other contexts?**

The implications of the study for clinical practice are considered, discussing their relevance in a broader context
