# Supplementary material for: Barriers and facilitators to successful management of type 2 diabetes mellitus in Latin America and the Caribbean: A systematic review
Source: PLoS One. 2020 Sep 4;15(9):e0237542. doi: 10.1371/journal.pone.0237542 (PMC7473520; doi:10.1371/journal.pone.0237542)
Supplement: S1 Table — DM: Diabetes Mellitus, T2DM: Type 2 Diabetes Mellitus, HBP: High Blood Pressure, NCC: Neurocysticercosis; NA: Not Applicable, ND: No Data. (DOCX) [file pone.0237542.s004.docx]

# *S1 Table.* Characteristics of the 48 studies included (in detail).

|  |  |  |  |  |  | **Patients with Diabetes Mellitus** | | | | | |
| --- | --- | --- | --- | --- | --- | --- | --- | --- | --- | --- | --- |
| **First Author ^Ref^** | **Year** | **Study location** | **Study method** | **Disease treated** | **Participants (N)** | **N**  **Men** | **N**  **Women** | **Age**  mean±SD  -years- | **Economic status** | **Educational**  **level** | **Ethnicity** |
| Adams OP, 2010 ^27^ | 2010 | Barbados | Qualitative | T2DM  HBP | Health workers (83) | 14 | 69 | Range:  [34 – 49] | NR | NR | NR |
| Adams OP, 2011^28^ | 2011 | Barbados | Qualitative | T2DM  HBP | Patients (16 DM/21) | 3 | 13 | 59 | NR | NR | NR |
| Albarran NB ^29^ | 2006 | México | Mixed | T2DM | Patients (49) Relatives (38) | 6 | 43 | 55.8 ± 11 | Middle- and low-income | Elementary (mean: 4.5 ± 3.4) | NR |
| Andrews CM ^30^ | 2018 | Guatemala | Qualitative | T2DM | Patients (55) Health workers (10) Traditional healers (8) | 5 | 50 | 53.3±11.4 | NR | NR | Indigenous |
| Ávila-Sansores GM^31^ | 2013 | México | Qualitative | T2DM  HBP | Patients (7 DM/ 9) | 2 | 5 | Range:  [42 – 67] | Low-income | Illiterate: 77.8% | NR |
| Bermeo-Cabrera J ^32^ | 2018 | México | Quantitative | T2DM | Patients (200) | 70 | 130 | 61.5 ± 12 | NR | Elementary ≤ 30% | NR |
| Bersusa AA ^33^ | 2010 | Brazil | Quantitative | DM  HBP | Patients (342 DM / 4,379 population) | ND | ND | The widest range ≥ 60 | NR | NR | NR |
| Cardenas MK ^34^ | 2016 | Peru | Mixed | DM | Patients (39) Health workers (60) | ND | ND | ND | NR | NR | NR |
| Carvalho SL ^35^ | 2018 | Brazil | Qualitative | T2DM | Patients (53) Caregivers (19) | NR | NR | ≥ 60 yrs | NR | NR | NR |
| Castro B ^36^ | 2018 | Dominican Republic | Quantitative | DM  HBP | Patients (16 DM / 149) | NR | NR | NR | Low-income | NR | NR |
| Chary A ^37^ | 2012 | Guatemala | Mixed | T2DM | Patients (103) Health workers (3) Community leaders (3) | 17 | 86 | 54.9 ± 12.7 | NR | Elementary | Mostly indigenous Maya |
| Dekker AM ^38^ | 2017 | Belize | Mixed | DM  HBP | Charts (112 DM/ 178)  Patients (19 DM/ 25) Health workers (20) | 29 | 83 | 59 ± 19 | NR | Elementary:84% | Mostly indigenous people |
| Eliaschewitz FG ^39^ | 2018 | Brazil | Mixed | T2DM | Health workers (6) | NA | NA | NA | NA | NA | NA |
| Faria CCC ^40^ | 2013 | Brazil | Quantitative | DM | Patients (187) | 33 | 154 | 57.8% > 60 yrs | 71.6% < 3 minimum wages | Incomplete elementary:57.6% (26.6% illiterate) | NR |
| Fort MP, 2011 ^41^ | 2013 | México, Costa Rica | Qualitative | T2DM  HBP | Patients (70) | NR | NR | Range:  [30 -73] | NR | NR | NR |
| Fort MP, 2015 ^42^ | 2016 | Mexico | Qualitative | T2DM  HBP | Patients (9) Relatives (6) Health workers (9) | 9 | 0 | Range:  [47 – 69] | NR | NR | NR |
| da Gama CAP ^43^ | 2017 | Brazil | Qualitative | DM | Health workers and other stakeholders (20) | NA | NA | NA | NA | NA | NA |
| García Castro M ^44^ | 2005 | Cuba | Quantitative | DM | Patients (68 T2DM/81) | 37 | 44 | 63% range:  [25–59] | NR | Elementary:14.8% Secondary: 27.2% | NR |
| Goldín L ^45^ | 2017 | Guatemala | Qualitative | T2DM | Patients (36 DM/39) Health workers (60) Health directors (9) Stakeholders (3) | 3 | 31 | 51.5 ± 7.3 | Low-income | Elementary | Indigenous Maya |
| Gouveia BdLA (a)^46^ | 2020 | Brazil | Mixed | T2DM | Patients (32) | NR | NR | NR | 56.5 [39-77] | Incomplete elementary:43.7% | Not white: 65.6% |
| Gouveia BdLA (b)^47^ | 2020 | Brazil | Mixed | T2DM | Patients (32) | NR | NR | NR | 59.06±11.08 | Incomplete elementary:50% | Not white: 62.5% |
| Henrique NN ^48^ | 2008 | Brazil | Quantitative | DM  HBP | Patients (25 DM/ 73) | 7 | 18 | 41.1% > 60 yrs | NR | NR | NR |
| Juárez-Ramírez C ^49^ | 2019 | Mexico | Mixed | T2DM | Patients (195) | 27 | 168 | 56.5 | NR | Illiterate: 39% | Indigenous Maya |
| Lagunes-Córdoba R^50^ | 2017 | México | Qualitative | T2DM | Health workers (18) | NA | NA | NA | NA | NA | NA |
| Lenz R ^51^ | 2010 | Chile | Mixed | T2DM | Health workers (88) | NA | NA | NA | NA | NA | NA |
| Lerman I ^52^ | 2009 | México | Quantitative | T2DM | Patients (29) | 11 | 18 | 59 ± 8 | Low-income | Elementary | NR |
| de Lima Santos ^53^ | 2018 | Brazil | Qualitative | T2DM | Patients (26) | 17 | 9 | Range:  [38 – 64] | NR | NR | NR |
| Lopez Stewart G ^54^ | 2007 | Argentina, Brazil, Chile, Costa Rica, Ecuador, Guatemala, Mexico,Peru,  Venezuela | Quantitative | T2DM | Patients (3,451) Health workers (377) | 1,597 | 1,844 | 59.7 ±10.5 | Private clinic | NR | NR |
| Medina Fernández J^55^ | 2018 | México | Qualitative | T2DM | Patients (8) | 2 | 6 | Range:  [39 - 72] | Low-income | Illiterate: 62.5% | Indigenous Maya |
| Moura PC ^56^ | 2018 | Brazil | Quantitative | DM | Patients (77) | 30 | 47 | 57.4 ±11.7 | 72.7% < 3 min. wages | Incomplete elementary: 61% | NR |
| Nieblas-Bedolla E ^57^ | 2019 | Guatemala | Qualitative | T2DM | Patients (15) | 7 | 8 | 46.8 | Low-income | NR | Indigenous |
| Oliveira DM ^58^ | 2016 | Brazil | Qualitative | DM | Health workers (18) | NA | NA | NA | NA | NA | NA |
| Oliveira NF ^59^ | 2011 | Brazil | Qualitative | DM | Patients (16) | NR | NR | NR | NR | NR | NR |
| Parra DI ^60^ | 2019 | Colombia | Quantitative | T2DM  HBP | Patients (153 T2DM/ 500) | 31% | 69% | Median [Q1-Q3]: 68 [59-75] | Low-income: 58% | Elementary: 65% | NR |
| Péres DS, 2006 ^61^ | 2006 | Brazil | Qualitative | T2DM | Patients (8) | 0 | 8 | Range: 49-76 | Mostly < 2 minimum wages | Incomplete elementary | NR |
| Péres DS, 2007 ^62^ | 2007 | Brazil | Qualitative | T2DM | Patients (24) | NR | NR | Range: 25-76 | NR | Mostly elementary | NR |
| Péres DS, 2008 ^63^ | 2009 | Brazil | Qualitative | T2DM | Patients (8) | 0 | 8 | Range: 49-76 | 62.5% < 2 minimum wages. | Elementary | NR |
| Perez-Leon S ^64^ | 2018 | Peru | Qualitative | T2DM  + other diseases | Patients (4 DM/ 16)  Relatives (13)  Community dwellers (119) | NR | NR | NR | In poverty: 73% | NR | NR |
| Pesantes MA ^65^ | 2019 | Peru | Qualitative | T2DM | Patients (20) | 5 | 15 | 62.53 | Low-income | NR | NR |
| Pesantes MA ^66^ | 2020 | Peru plus non-LAC countries | Qualitative | DM  HBP | Patients (5 DM/11)  Caregivers (3)  Head of household (5) | 1 | 4 | NR | NR | NR | NR |
| Pinzón-Rocha M ^67^ | 2013 | Colombia | Qualitative | T2DM | Caregivers (15) | NA | NA | NA | NA | NA | NA |
| Quintana AA ^68^ | 2008 | Chile | Quantitative | T2DM | Patients (128) | 38 | 90 | NR | The widest range > 60 | Elementary:67.7% (9.4% illiterate) | NR |
| Rodríguez Salvá A^69^ | 2019 | Cuba | Quantitative | T2DM | Patients (580) | 209 | 371 | Median [range]:  65 [19-96] | NR | Middle / high education: 85.4% | Not white: 45.5% |
| Rodríguez-Morán M^70^ | 1997 | México | Quantitative | T2DM | Patients (82) | 25 | 57 | 58.9 ±9.2  (Group 1) 57.8 ± 9.7 (Group 2) | Unemployed: 69.5% | Elementary: (mean: 4 yrs) | NR |
| Roopnarinesingh N^71^ | 2015 | Trinidad and Tobago | Quantitative | DM | Health workers (≈100) | NA | NA | NA | NA | NA | NA |
| Salci MA ^72^ | 2017 | Brazil | Qualitative | DM | Health workers (35) Health gestors (3) | NA | NA | NA | NA | NA | NA |
| Santos AFL ^73^ | 2011 | Brazil | Qualitative | T2DM | Patients (20) | 2 | 18 | Range: 30-59 | 85% ≤ 3  min. wages | Elementary 70% Secondary 30% | Non-indigenous |
| Santos ECB ^74^ | 2005 | Brazil | Qualitative | T2DM  HBP | Patient (1)  Caregiver (1) | 0 | 1 | 52 yrs. | NR | Elementary | NR |
| Souza MLP ^75^ | 2008 | Brazil | Qualitative | DM  HBP | Health workers (ND) | NA | NA | NA | NA | NA | NA |
| de Souza Rocha NB^76^ | 2019 | Brazil | Qualitative | T2DM | Patients (20) | NR | NR | ≥ 60 yrs. | NR | NR | NR |
| Stacciarini TSG ^77^ | 2008 | Brazil | Quantitative | DM | Patients (269) | 77 | 192 | 61% > 60 yrs. | NR | Elementary: 89.2% (34.2% illiterate) | NR |
| Taylor CG ^78^ | 2017 | Barbados | Quantitative | T2DM | Patients (117) | 37 | 80 | Mean: 66 ± 13 | NR | Secondary: 65% | Black 90% |
| Teston EF ^79^ | 2017 | Brazil | Qualitative | T2DM | Patients (18) | 9 | 9 | Range:  [40-69] | Mostly 2 minimum wages | Elementary: range 4-7 yrs | NR |
| Torres HC, 2011 ^80^ | 2011 | Brazil | Qualitative | T2DM | Patients (12) | 3 | 9 | 60.9 ± 8.4 | 1-3 minimum wages | Incomplete elementary: 74.1% | NR |
| Torres HC, 2010 ^81^ | 2010 | Brazil | Qualitative | T2DM | Health workers (23) | NA | NA | NA | NA | NA | NA |
| Vencio S ^82^ | 2017 | Brazil + non-LAC countries | Quantitative | T2DM | Patients (100) Health workers (55) | 60 | 40 | 52.4 | Low-income:  8% | NR | NR |
| Vigeta SMG ^83^ | 2014 | Brazil | Qualitative | DM | Patients (6) | 6 | 0 | Range:  [60-75] | NR | Elementary: 50% Higher: 50% | NR |
| Whittemore R ^84^ | 2019 | Mexico | Qualitative | T2DM | Patients (20)  Health workers (19) | 4 | 16 | 52.5 ± 9.9 | Financial insecurity:90% | Elementary ≤55% | NR |
| Wint YB ^85^ | 2006 | Jamaica | Mixed | DM | Patients (133) | 35 | 98 | Range: [20–90] | NR | Mostly elementary education: 67% | NR |
| Yoshida VC ^86^ | 2016 | Brazil | Qualitative | DM  HBP | Patients (7 DM/ 10) | 7 | 0 | 43.4 | NR | Range: 2-10 yrs | NR |

NA: Not Applicable; ND: Not Reported; DM: Diabetes Mellitus; T2DM: Type 2 Diabetes Mellitus; HBP: High Blood Pressure; LAC: Latin And Caribbean
