# Supplementary material for: Barriers and facilitators to successful management of type 2 diabetes mellitus in Latin America and the Caribbean: A systematic review
Source: PLoS One. 2020 Sep 4;15(9):e0237542. doi: 10.1371/journal.pone.0237542 (PMC7473520; doi:10.1371/journal.pone.0237542)
Supplement: S2 Table — NA: Not Applicable (DOCX) [file pone.0237542.s005.docx]

# S2 Table. Critical appraisal assessment in detail.

| **First Author** | Q1 | Q2a | Q2b | Q2c | Q3 | Q4 | Q5 |
| --- | --- | --- | --- | --- | --- | --- | --- |
| Adams OP, 2010 | YES | UNCLEAR |  | UNCLEAR | YES | YES | NO |
| Adams OP, 2011 | YES | UNCLEAR |  | YES | YES | YES | YES |
| Albarran NB | YES | YES | NO | NO | YES | YES | YES |
| Andrews CM | YES | YES |  | UNCLEAR | YES | YES | YES |
| Ávila Sansores GM | YES | UNCLEAR |  | YES | UNCLEAR | YES | YES |
| Bermeo-Cabrera J | YES | YES | UNCLEAR |  | YES | YES | YES |
| Bersusa AA | UNCLEAR | UNCLEAR | YES |  | YES | YES | YES |
| Cardenas MK | YES | NO | NO | NO | UNCLEAR | YES | YES |
| Carvalho SL | YES | YES | UNCLEAR |  | NO | YES | YES |
| Castro B | YES | NO | NO |  | YES | YES | YES |
| Chary A | YES | YES | YES | NO | UNCLEAR | YES | YES |
| Dekker AM | YES | NO | NO | UNCLEAR | UNCLEAR | YES | YES |
| Eliaschewitz FG | YES | NO | NO | NO | YES | YES | YES |
| Faria CCC | YES | YES | YES |  | UNCLEAR | YES | YES |
| Fort MP, 2011 | YES | YES |  | NO | YES | YES | UNCLEAR |
| Fort MP, 2015 | YES | NO |  | NO | YES | YES | YES |
| García Castro M | YES | UNCLEAR | NO |  | UNCLEAR | YES | YES |
| Goldín L | YES | UNCLEAR |  | UNCLEAR | YES | YES | YES |
| Henrique NN | YES | UNCLEAR | NO |  | NO | NO | NO |
| Lagunes-Córdoba R | YES | NO |  | NO | YES | YES | YES |
| Lenz R | YES | UNCLEAR | NO | NO | YES | YES | YES |
| Lerman I | YES | YES | UNCLEAR |  | UNCLEAR | YES | YES |
| Lopez Stewart G | YES | UNCLEAR |  | YES | YES | NO | UNCLEAR |
| Medina Fernández J | YES | UNCLEAR |  | UNCLEAR | YES | YES | YES |
| Moura PC | YES | UNCLEAR | UNCLEAR |  | UNCLEAR | YES | YES |
| Oliveira DM | YES | YES |  | YES | YES | YES | UNCLEAR |
| Oliveira NF | YES | NO |  | UNCLEAR | YES | YES | NO |
| Péres DS, 2006 | YES | YES |  | YES | YES | YES | YES |
| Péres DS, 2007 | YES | UNCLEAR |  | UNCLEAR | YES | YES | UNCLEAR |
| Péres DS, 2008 | UNCLEAR | UNCLEAR |  | YES | YES | YES | YES |
| Perez-Leon S | YES | YES |  | UNCLEAR | YES | YES | YES |
| Pinzón-Rocha M | YES | YES |  | YES | YES | YES | UNCLEAR |
| Quintana AA | UNCLEAR | NO | UNCLEAR |  | YES | UNCLEAR | YES |
| Rodríguez-Morán M | YES | NO | UNCLEAR |  | UNCLEAR | YES | NO |
| Roopnarinesingh N | YES | YES | NO |  | YES | YES | UNCLEAR |
| Salci MA | YES | UNCLEAR |  | UNCLEAR | YES | YES | YES |
| Santos AFL | YES | YES |  | UNCLEAR | YES | YES | YES |
| Santos ECB | YES | YES |  | NO | NO | YES | UNCLEAR |
| Souza MLP | NO | NO |  | UNCLEAR | YES | YES | NO |
| Stacciarini TSG | YES | YES | NO |  | UNCLEAR | YES | YES |
| Taylor CG | UNCLEAR | YES | NO |  | UNCLEAR | NO | YES |
| Teston EF | YES | UNCLEAR |  | YES | YES | YES | YES |
| Torres HC, 2011 | YES | YES |  | YES | YES | YES | YES |
| Torres HC, 2010 | YES | UNCLEAR |  | NO | YES | YES | UNCLEAR |
| Vencio S | YES | NO | NO |  | YES | UNCLEAR | UNCLEAR |
| Vigeta SMG | YES | NO |  | YES | YES | YES | NO |
| Wint YB | YES | YES | YES | UNCLEAR | YES | NO | YES |
| Yoshida VC | YES | UNCLEAR |  | YES | YES | YES | YES |
| Gouveia BdLA, 2020a | YES | YES | NO | YES | UNCLEAR | NO | UNCLEAR |
| Gouveia BdLA, 2020b | YES | YES | NO | YES | UNCLEAR | NO | YES |
| Rodríguez Salvá A | YES | YES | UNCLEAR | NA | NO | YES | NO |
| Whittemore R | YES | YES | NA | YES | YES | YES | YES |
| da Gama CAP | YES | YES | NA | YES | YES | YES | YES |
| Pesantes MA, 2020 | YES | YES | NA | UNCLEAR | YES | YES | YES |
| Parra DI | YES | NO | UNCLEAR | NA | UNCLEAR | UNCLEAR | NO |
| Pesantes MA, 2019 | YES | YES | NA | NO | YES | YES | YES |
| Nieblas-Bedolla E | YES | YES | NA | UNCLEAR | YES | YES | UNCLEAR |
| de Souza Rocha NB | YES | YES | NA | YES | YES | YES | UNCLEAR |
| Juárez-Ramírez C | YES | NO | NO | NO | YES | YES | NO |
| De Lima Santos | YES | YES | NA | NO | YES | YES | YES |
