# Supplementary material for: Barriers and facilitators to successful management of type 2 diabetes mellitus in Latin America and the Caribbean: A systematic review
Source: PLoS One. 2020 Sep 4;15(9):e0237542. doi: 10.1371/journal.pone.0237542 (PMC7473520; doi:10.1371/journal.pone.0237542)
Supplement: S3 Table — NA: Not Applicable (DOCX) [file pone.0237542.s006.docx]

***S3 Table*. Barriers and facilitators to diabetes care from the perspective of patients, caregivers, relatives and community members.**

| **Domain (n studies)** | **Theme** | **N** | **Examples of Barriers Identified** | N | **Example of Facilitators Identified** |
| --- | --- | --- | --- | --- | --- |
| **Environmental context and resources (30)** | Health system context | 14 | - Lack of health insurance or health care access - Shortage of physical resources - Lack of human resources - Organizational weaknesses | 7 | - Good insurance coverage and heath access - Strong organizational structure - Multidisciplinary teams - Sufficient human resources |
|  | Patient context | 28 | - Financial issues - Work constraints | 5 | - Financial security |
|  | Environmental context | 7 | - Weather conditions - Lack of green spaces/ urban infrastructure/ security - Long distance to appointments - Lack of healthy food at workplace | 0 | - Taxing and labelling beverages and food - Providing sidewalks and cycling lanes |
| **Social influences (26)** | (Lack of) Support from family or friends | 15 | - Lack of support related to diet at home and, also, absence of family | 16 | - Support from family / friends to follow diet, translate language, inject insulin, and economical support |
|  | Social gatherings | 7 | - Social pressure to disrupt diet - No convenient food or beverages at social gatherings | 1 | - Avoiding social gatherings |
|  | Stigma | 4 | - Stigma surrounding illness or use of insulin | 0 | NR |
|  | Peer support | 0 | NR | 7 | - Peers support and meeting groups |
| **Social professional role and identity (23)** | Health professionals’ role | 16 | - Paternalistic attitude and vertical communication - No patient-centred recommendations | 9 | - Direct communication with patient - Patient-centered recommendations - Relevant educational role of nurses |
|  | Patients identity | 9 | - Denial or non-acceptance of the disease | 0 | NR |
|  | Gender role of men | 4 | - Prioritising job - Men must be strong - High alcohol and tobacco consumption | 0 | NR |
|  | Gender role of women | 2 | - Prioritising taking care of others - Non decision-making power over self-care | 0 | NR |
| **Beliefs about consequences (18)** | (Dis) trust | 7 | - Medication, tests, and doctor’s advice will not work or it is not necessary | 4 | Trust in medication and doctor advise |
|  | Injecting insulin | 2 | - Avoiding starting insulin because it is considered worse   - Taking oral medication for granted | 0 | NR |
|  | Disease severity | 0 | NR | 6 | Awareness of disease severity |
|  | Home remedies | 9 | - Trust in home remedies as medication substitutes | 3 | Availability of potentially effective home remedies when economic issues prevent adherence to pharmaceuticals |
| **Behavioural regulation (17)** | Following a diet or exercise routine | 17 | - Loss of control on the impulse of eating  - Lack of motivation  - Diet is monotonous, unfilled, imposed, not fitting preferences and disrupting to daily routine | 0 | NR |
|  | Comorbidities and polypharmacy | 7 | - Comorbidities or complications impeding exercise  - Vision problems reduce capacity to inject insulin | 0 | NR |
|  | Strategies to control glycaemia | 0 | NR | 2 | - Planning daily routine around injecting insulin - Monitoring glycaemia before and after exercise |
| **Emotion (17)** | Emotional burden of disease | 15 | - Fear of side effects, tests and injecting - Depression or stress - Punishment or shame related to insulin - - Feeling of loss of independence | 3 | - Fear of death or some complications  - No fear of hypoglycaemia  Being calm |
| **Knowledge (16)** | Patient knowledge | 13 | - Low health literacy   Bad experience of a family member | 2 | - Mass media providing educational messages   - Learning from family experience |
|  | Professional knowledge | 2 | Insufficient knowledge to manage side effects and communicate with patient | 1 | - Updated training provided to health providers |
| **Reinforcement (12)** | (Lack of) symptoms | 8 | - Side effects of medication, also hypoglycaemia   - Absence of symptoms | 10 | - Getting better after following recommendations   - Presence of pain or symptoms |
| **Optimism (6)** | Patient faith | 1 | - Faith in God | 5 | - Faith in God  - Belief in being cured |
|  | Professional attitude | 0 | NR | 0 | - Positive attitude of health professionals |
| **Skills (4)** | Abilities to manage the disease | 4 | - Unable to control diet and cook proper meals - Unable to inject insulin and self-monitoring of blood glucose | 0 | NR |
| **Memory, attention, and decision processes (4)** | Following medication | 4 | - Forgetfulness - Frequent medication or advise changes | 0 | NR |
| **Intentions (4)** | Changing patient habits | 2 | - No intention of following diet or exercise recommendations | 2 | - Keeping healthy |
|  | Professional training | 0 | NR | 0 | - Successfully completing health professionals training |
| **Beliefs about capabilities (1)** | Being capable of controlling the disease | 1 | - Inability to change habits or control food intake   Not injecting insulin correctly | 0 | NR |

NR: Not Reported
