# Supplementary material for: Barriers and facilitators to successful management of type 2 diabetes mellitus in Latin America and the Caribbean: A systematic review
Source: PLoS One. 2020 Sep 4;15(9):e0237542. doi: 10.1371/journal.pone.0237542 (PMC7473520; doi:10.1371/journal.pone.0237542)
Supplement: S4 Table — NA: Not Applicable (DOCX) [file pone.0237542.s007.docx]

***S4 Table.* Barriers and facilitators to diabetes care from the perspective of the health professionals, health directors and other stakeholders.**

| **Domain (n studies)** | **Theme** | N | **Examples of Barriers Identified** | N | **Example of Facilitators Identified** |
| --- | --- | --- | --- | --- | --- |
| **Environmental context and resources (15)** | Health system context | 13 | - Lack of health insurance or health care access - Shortage of physical resources - Lack of human resources - Organizational weaknesses | 8 | - Good insurance coverage and heath access - Strong organizational structure - Multidisciplinary teams - Sufficient human resources |
|  | Patient context |  | - Financial issues - Work constraints | 3 | - Financial security |
|  | Environmental context | 12 | - Weather conditions - Lack of green spaces/ urban infrastructure/ security - Long distance to appointments - Lack of healthy food at workplace | 1 | - Taxing and labelling beverages and food - Providing sidewalks and cycling lanes |
| **Social professional role and identity (11)** | Health professionals’ role | 2 | - Paternalistic attitude and vertical communication - No patient-centred recommendations | 1 | - Direct communication with patient - Patient-centered recommendations - Relevant educational role of nurses |
|  | Patients identity | 10 | - Denial or non-acceptance of the disease | 0 | NR |
|  | Gender role of men | 4 | - Prioritising job - Men must be strong - High alcohol and tobacco consumption | 0 | NR |
|  | Gender role of women | 2 | - Prioritising taking care of others - Non decision-making power over self-care | 0 | NR |
| **Knowledge (11)** | Patient knowledge | 0 | - Low health literacy - Bad experience of a family member | 1 | - Mass media providing educational messages   - Learning from family experience |
|  | Professional knowledge | 4 | - Insufficient knowledge to manage side effects and communicate with patient | 4 | - Updated training provided to health providers |
| **Social influences (8)** | (Lack of) Support from family or friends | 1 | - Lack of support related to diet at home and, also, absence of family | 5 | - Support from family / friends to follow diet, translate language, inject insulin, and economical support |
|  | Social gatherings | 5 | - Social pressure to disrupt diet - No convenient food or beverages at social gatherings | 0 | - Avoiding social gatherings |
|  | Stigma | 1 | - Stigma surrounding illness or use of insulin | 0 | NR |
|  | Peer support | 3 | NR | 2 | - Peers support and meeting groups |
| **Behavioural regulation (6)** | Following a diet or exercise routine | 6 | - Loss of control on the impulse of eating  - Lack of motivation  - Diet is monotonous, unfilled, imposed, not fitting preferences and disrupting to daily routine | 0 | NR |
|  | Comorbidities and polypharmacy | 3 | - Comorbidities or complications impeding exercise  - Vision problems reduce capacity to inject insulin | 0 | NR |
|  | Strategies to control glycaemia | 3 | NR | 1 | - Planning daily routine around injecting insulin - Monitoring glycaemia before and after exercise |
| **Beliefs about consequences (5)** | (Dis) trust | 0 | - Medication, tests and doctor’s advice will not work or it is not necessary | 0 | - Trust in medication and doctor advise |
|  | Injecting insulin | 2 | - Avoiding starting insulin because it is considered worse   - Taking oral medication for granted | 0 | NR |
|  | Disease severity | 1 | NR | 0 | - Awareness of disease severity |
|  | Home remedies | 0 | - Trust in home remedies as medication substitutes | 2 | - Availability of potentially effective home remedies when economic issues prevent adherence to pharmaceuticals |
| **Emotion (5)** | Emotional burden of disease | 3 | - Fear of side effects, tests and injecting - Depression or stress - Punishment or shame related to insulin   - Feeling of loss of independence | 0 | - Fear of death or some complications  - No fear of hypoglycaemia   - Being calm |
| **Reinforcement (3)** | (Lack of) symptoms | 5 | - Side effects of medication, also hypoglycaemia   - Absence of symptoms | 2 | - Getting better after following recommendations   - Presence of pain or symptoms |
| **Optimism (3)** | Patient faith | 3 | - Faith in God | 2 | - Faith in God  - Belief in being cured |
|  | Professional attitude | 0 | NR | 1 | - Positive attitude of health professionals |
| **Memory, attention, and decision processes (2)** | Following medication | 1 | - Forgetfulness - Frequent medication or advise changes | 0 | NR |
| **Skills (1)** | Abilities to manage the disease | 0 | - Unable to control diet and cook proper meals - Unable to inject insulin and self-monitoring of blood glucose | 0 | NR |
| **Beliefs about capabilities (1)** | Being capable of controlling the disease | 2 | - Inability to change habits or control food intake - Not injecting insulin correctly | 0 | NR |
| **Intentions (1)** | Changing patient habits | 1 | - No intention of following diet or exercise recommendations | 0 | - Keeping healthy |
|  | Professional training | 0 | NR | 1 | - Successfully completing health professionals training |

NP: Not Reported
